# High-flow nasal cannula therapy as apneic oxygenation during endotracheal intubation in critically ill patients in the intensive care unit: a systematic review and meta-analysis

**DOI:** 10.1038/s41598-020-60636-9

**Published:** 2020-02-26

**Authors:** Hong-Jie Jhou, Po-Huang Chen, Chin Lin, Li-Yu Yang, Cho-Hao Lee, Chung-Kan Peng

**Affiliations:** 1 Division of General Practice, Department of Medical Education, Changhua Christian Hospital, Changhua, Taiwan, ROC; 2 Department of Internal Medicine, Tri-Service General Hospital, National Defense Medical Center, Taipei, Taiwan, ROC; 3 School of Public Health, National Defense Medical Center, Taipei, Taiwan, ROC; 4 Department of Research and Development, National Defense Medical Center, Taipei, Taiwan, ROC; 5 Department of Neurology, Changhua Christian Hospital, Changhua, Taiwan, ROC; 6 Division of Hematology and Oncology Medicine, Department of Internal Medicine, Tri-Service General Hospital, National Defense Medical Center, Taipei, Taiwan, ROC; 7 Division of Pulmonary and Critical Care Medicine, Department of Internal Medicine, Tri-Service General Hospital, National Defense Medical Center, Taipei, Taiwan, ROC

**Keywords:** Respiratory distress syndrome, Hypoxia, Outcomes research

## Abstract

We conducted a systematic review and meta-analysis to assess the clinical efficacy of high-flow nasal cannula (HFNC) therapy as apneic oxygenation in critically ill patients who require endotracheal intubation in the intensive care unit (ICU). This systematic review and meta-analysis included six randomized controlled trials and a prospective study identified in PubMed, Embase, Cochrane Library, and the Web of Science until August 18, 2019. In this meta-analysis including 956 participants, HFNC was noninferior to standard of care during endotracheal intubation regarding incidence of severe hypoxemia, mean lowest oxygen saturation, and in-hospital mortality. HFNC significantly shortened the ICU stay by a mean of 1.8 days. In linear meta-regression interaction analysis, the risk ratio of severe hypoxemia decreased with increasing baseline partial oxygen pressure (PaO_2_) to fraction of inspired oxygen (FiO_2_) ratio. In subgroup analysis, HFNC significantly reduced the incidence of severe hypoxemia during endotracheal intubation in patients with mild hypoxemia (PaO_2_/FiO_2_> 200 mmHg; risk difference, −0.06; 95% confidence interval, −0.12 to −0.01; number needed to treat = 16.7). In conclusion, HFNC was noninferior to standard of care for oxygen delivery during endotracheal intubation and was associated with a significantly shorter ICU stay. The beneficial effect of HFNC in reducing the incidence of severe hypoxemia was observed in patients with mild hypoxemia.

## Introduction

Each year in the United States, approximately 1.5 million patients are estimated to receive endotracheal intubation and the rate of intubation is increasing in the hospital^1^. Hypoxemia, a frequently reported complication of intubation, is considered a predisposing factor for cardiac arrest and death^2–5^. Therefore, oxygenation during endotracheal intubation plays an important role in prolonging the maintenance of acceptable oxygen saturation levels.

In 1959, Frumin *et al*.^6^ were the first to develop apneic oxygenation, which delivered supplemental oxygen via nasal cannulation during surgery and anesthesia to allow for sustained levels of sufficient oxygen in alveoli and blood. A recent clinical trial convincingly demonstrated that apneic oxygenation during endotracheal intubation reduced the incidence of hypoxemia while increasing first-pass success rate and peri-intubation oxygen saturation^7^. Nevertheless, conventional oxygen therapy, which utilizes nasal cannulas, simple face masks, or Venturi masks for oxygenation, is sometimes ineffective in critically ill patients, especially in those with hypoxemia^8^.

High-flow nasal cannula (HFNC) is a novel respiratory management strategy that delivers humidified and warm supplemental oxygen at flow rates of up to 60 L/min in adults. Compared to standard of care oxygen therapy, HFNC has several physiological advantages, including increased positive end-expiratory pressure (PEEP), and constant fraction of inspired oxygen (FiO_2_)^9^. Due to the ease of setting, tolerance, and effectiveness, HFNC is a widely used in patients in the intensive care unit (ICU) for hypoxemic respiratory failure^10^.

Consequently, HFNC as apneic oxygenation during endotracheal intubation is proposed to be beneficial in preventing hypoxemia. Miguel-Montanes *et al*.^11^ reported that HFNC led to a significant reduction in the incidence of severe hypoxemia (peripheral capillary oxygen saturation [SpO_2_] < 80%) compared with the bag-valve mask during intubation (2% vs 14%). Similarly, Vourc’h *et al*.^12^ found that the lowest oxygen saturation level was higher with HFNC. Despite these encouraging benefits of HFNC, the latest randomized control trial (RCT)^13^ by Frat *et al*. reported that HFNC did not significantly alter the risk of severe hypoxemia during intubation.

RCTs reported contradictory outcomes with HFNC as apneic oxygenation, and there is currently no consensus regarding whether the rate of hypoxemia is lower with HFNC during endotracheal intubation than with standard of care oxygen treatment. Therefore, there is an urgent need for evidence synthesis based on the comparison of HFNC with standard of care during intubation in critically ill patients.

## Methods

### Data sources and searches

We performed a comprehensive search without language restrictions using PubMed, Embase, Cochrane Library, and the Web of Science to identify studies that assessed the outcomes of HFNC as apneic oxygenation during endotracheal intubation in critically ill patients, including those with hypoxemic respiratory failure, those in a comatose stage, and those with hemodynamic dysfunction, in the setting of ICU.

Two independent investigators (P.H.C. and H.J.J.) conducted a systematic search using the terms “high-flow nasal cannula”, “apneic oxygenation”, and “intubation” and utilized medical subject headings or their equivalents and normal text keywords as search terms (Supplementary Information 1) until 18 August, 2019. Manual screening for references from original articles, previous systematic reviews, and conference abstracts was performed to identify eligible studies.

We adhered to the Preferred Reporting Items for Systematic Reviews and Meta Analyses (PRISMA) (Supplementary Information 2) and the Meta-analysis Of Observational Studies in Epidemiology (MOOSE) (Supplementary Information 3) guidelines for performing systematic reviews and meta analyses of RCTs and observational studies^14,15^. The protocol for this systematic review had registered with PROSPERO (CRD42019139408). The first or corresponding authors of the studies were contacted to provide additional information if required.

### Eligibility criteria and exclusion criteria

To be included in the analysis, the studies had to meet the following criteria: (1) study cohort comprising only adult patients requiring endotracheal intubation who were admitted to the ICU; (2) administration of HFNC cannula therapy, also known as trans-nasal humidified rapid-insufflation ventilatory exchange^16^ (oxygen delivery system comprising 100% humidified and heated oxygen at a flow rate > 15 L/min and up to 70 L/min) during the apneic period of endotracheal intubation, compared to standard of care (i.e., no management or oxygen administration by nasal cannulas, simple face masks, or Venturi masks during endotracheal intubation); (3) RCT or prospective non-randomized study.

The studies meeting the following criteria were excluded from the analysis: (1) endotracheal intubation performed in an out-of-hospital setting or in the operating room, (2) lack of reporting on outcomes of interest such as lowest oxygen saturation and number of desaturation events during endotracheal intubation.

Two reviewers (H.J.J. and P.H.C.) appraised all eligible citations independently and extracted various data into an electronic database from original manuscripts of eligible studies. In case of disagreement, the same authors consulted with another author (C.H.L.), the decisions were obtained after group discussion.

### Outcome measurement

The following outcomes were extracted: (1) major outcomes: incidence of severe hypoxemia (SpO_2_ < 80%) during the endotracheal intubation, mean lowest oxygen saturation during endotracheal intubation, ICU length of stay, and in-hospital mortality; (2) minor outcomes: incidence rates of mild hypoxemia (SpO_2_ < 90%) and life-threatening hypoxemia (SpO_2_ < 70%) during the endotracheal intubation, first-pass success (success on the first laryngoscopy attempt), duration of endotracheal intubation procedure period, shock (defined by systolic blood pressure <80 mmHg or that requiring vasopressor introduction or increasing vasopressor dose by more than 30%)^17^, cardiovascular complications (defined as shock, arrhythmia, and cardiac arrest)^18^, ventilator-associated pneumonia (pneumonia that occurs within 48–72 hours following mechanical ventilation)^19^, and duration of ventilation.

For dichotomous outcomes, we extracted the proportions in both the experimental and the comparator arms. For continuous outcomes, we extracted the number of participants as well as the mean values with the standard deviation for the outcome measurement per arm.

The quality of the RCTs (Supplementary Information 4) was appraised by H.J.J. and P.H.C using the Cochrane Handbook for Systematic Reviews of Interventions^20^. Furthermore, we assessed the quality of prospective non-randomized studies using the Newcastle-Ottawa Scale^21^. Any disagreement was resolved via group discussions^22^. Risk of bias graphs were generated using Review Manager 5.3 software^23^.

### Data synthesis and analysis

We conducted data analysis as recommended in the Cochrane Handbook for Systematic Reviews of Interventions^20^. We calculated dichotomous outcomes by conducting random-effects meta-analysis proposed by DerSimonian and Laird^24^ and the Mantel-Haenszel fixed-effects model^25^ using risk difference (RD) with 95% confidence interval (CI). To measure continuous outcomes, we employed the generic inverse variance method fixed-effect model and DerSimonian and Laird random-effects model^24^ meta-analysis using the mean difference (MD) approach with 95% CIs.

Heterogeneity was evaluated using the I square (I^2^) statistic and Cochran’s Q test. Statistically significant heterogeneity was defined as I^2^ > 50% and Cochran’s Q test *P* < 0.1^26^. We used a mixed-effects linear meta-regression model^27,28^ to evaluate the cause of heterogeneity for main outcomes, with variables including publication year, mean age, sex, and procedural variables including Body Mass Index (BMI), Simplified Acute Physiology Score (SAPS II), PaO_2_/FiO_2_ ratio, duration of intubation, and proceduralist expertise.

All statistical analyses were performed using the “metafor” and “meta”^29,30^ packages of R software version 3.3.1^31^. A *P* value < 0.05 with a two-tailed test indicated statistical significance without multiplicity correction in all analyses.

### Subgroup analysis and sensitivity analysis

Subgroup analysis was performed to detect clinical and statistical heterogeneity in cases of observable heterogeneity via the meta-regression test. We evaluated whether treatment effects on primary outcomes were robust by sensitivity analysis, which were performed based on the specific features of study design to explore the impact of excluding the prospective non-randomized study in meta-analysis.

## Results

**Figure 1:**
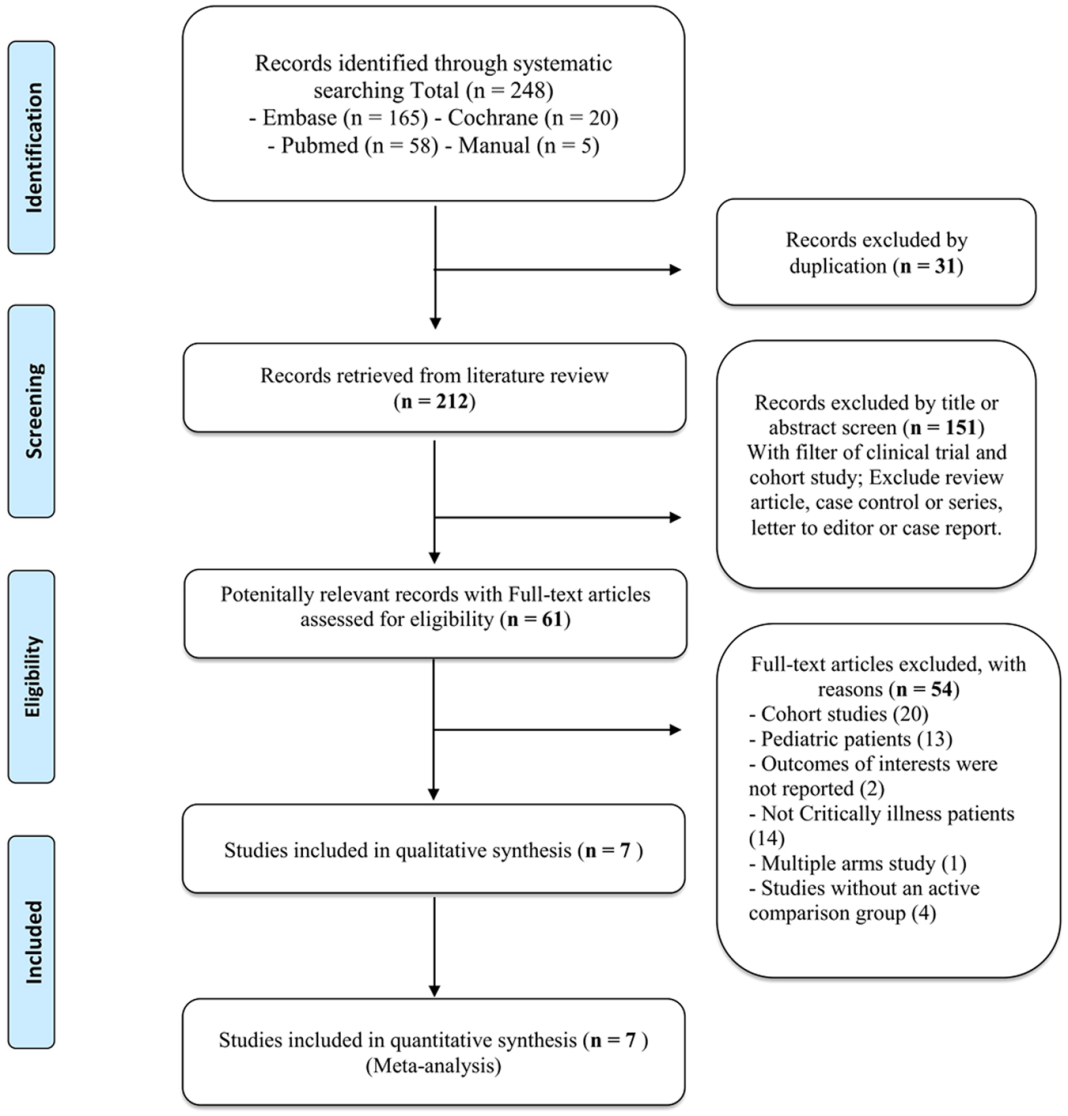
PRISMA flow diagram of the study selection: Flow diagram for the identification process for eligible studies.

A total of 243 articles were identified, and 212 articles remained after the removal of duplicates. After we screened the titles and abstracts, there were a total of 61 potentially associated articles. Ultimately, seven articles met the inclusion criteria for meta-analysis after the full-text review (Fig. 1) (Supplementary Information 5). Table 1 summarized the basic characteristics of the seven studies, including six RCTs and one prospective non-randomized study, which were published between 2015 and 2019^11–13,32–35^. There were a total of 956 participants, including 501 patients receiving HFNC during the apneic period and 455 receiving standard of care oxygen therapy.

**Table 1:** Basic characteristics of the included studies.

Author year	Trial names	Design (Country)	Population (Number)	Age (mean)	Male (%)	BMI (kg/m^2^)	PF ratio(PS)	Intervention	Comparator	Proceduralist Expertise
Miguel-Montanes 2015	NR	Prospective Study (France)	ICU patients with shock, AMS, or ARF (n = 101)	60 (years)	54.4	NR	NR (SAPS II: 45.5)	Pre-Ox: HFNC (60 L/min, FiO_2_ 100%) Ap-Ox: HFNC (60 L/min, FiO_2_ 100%)	Pre-Ox: NRB (15 L/min) Ap-Ox: nasopharyngeal catheter (6 L/min)	Trainees (Major)
Vourc’h 2015	PREOXYFLOW (NCT01747109)	RCT (France)	ICU patients with hypoxemic ARF (n = 119)	62.2 (years)	39.1	27.6	118 (SAPS II: 54.5)	Pre-Ox: HFNC (60 L/min, FiO 2 100%) Ap-Ox: HFNC (60 L/min, FiO_2_ 100%)	Pre-Ox: Face Mask (15 L/min oxygen flow) Ap-Ox: nil	Trainees (Major)
Jaber 2016	OPTINIV (NCT02530957)	RCT (France)	ICU patients with hypoxemic ARF (n = 49)	61 (years)	77.6	23.5	122 (SAPS II: 49)	Pre-Ox: HFNC (60 L/min, FiO_2_ 100%), NIV (PS of 10 cmH_2_O, PEEP of 5 cmH_2_O, FiO_2_ = 100%) Ap-Ox: HFNC (60 L/min, FiO_2_ 100%)	Pre-Ox: NIV (PS of 10 cmH_2_O, PEEP of 5 cmH_2_O, FiO_2_ = 100%) Ap-Ox: nil	Experts (Major)
Simon 2016	PV-4429 (NCT01994928)	RCT (Germany)	ICU patients with hypoxemic ARF (n = 40)	58.5 (years)	55.0	26.1	203 (SAPS II: 37)	Pre-Ox: HFNC (50 L/min, FiO_2_ 100%) Ap-Ox: HFNC (50 L/min, FiO_2_ 100%)	Pre-Ox: BVM without PEEP and pressure manometer (10 L/min) Ap-Ox: nil	Experts (All)
Semler 2016	FELLOW (NCT02051816)	RCT (US)	ICU patients with ARF or AMS (n = 150)	60 (years)	60.7	28.6	NR (APACHE II: 22)	Pre-Ox: NC, NRB, BVM, BiPAP Ap-Ox: HFNC (15 L/min, FiO_2_ 100%)	Pre-Ox: NC, NRB, BVM, BiPAP Ap-Ox: nil	Trainees (Major)
Guitton 2019	PROTRACH (NCT02700321)	RCT (France)	ICU patients with ARF or AMS (n = 184)	60.5 (years)	69.0	26.5	346 (SAPS II: 43.1)	Pre-Ox: HFNC (60 L/min, FiO_2_ 100%) Ap-Ox: HFNC (60 L/min, FiO_2_ 100%)	Pre-Ox: BVM (15 L/min) Ap-Ox: nil	Trainees (Major)
Frat 2019	FLORALI-2 (NCT02668458)	RCT (France)	ICU patients with ARDS (n = 313)	64 (years)	67.7	27	145 (SAPS II: 51.5)	Pre-Ox: HFNC (60 L/min, FiO_2_ 100%) Ap-Ox: HFNC (60 L/min, FiO_2_ 100%)	Pre-Ox: Face Mask (PEEP of 5 cmH_2_O, FiO_2_ = 100%) Ap-Ox: nil	Experts (Major)

AMS: altered mental status, ARDS: acute respiratory distress syndrome, ARF: acute respiratory failure, Ap-Ox: apneic oxygenation, BiPAP: biphasic positive airway pressure, BVM: bag-valve mask, DoI: duration of intubation, FiO_2_: fraction of inspired oxygen, HFNC: high flow nasal cannula, ICU: intensive care unit, NC: nasal cannula, NIV: non-invasive ventilation, NR: not reported, NRB: non-rebreathing mask, PEEP: positive end-expiratory pressure, Pre-Ox: pre-oxygenation, PS: physiologic score, RCT: randomized control trial, US: United State.

### Major outcomes (Summary of major findings were described at Supplementary Information 6)

**Figure 2:**
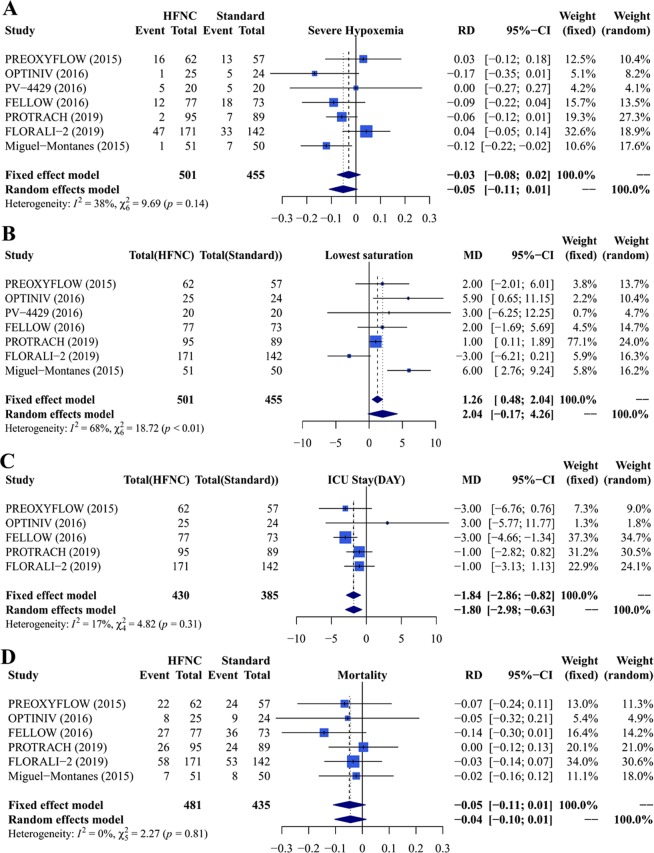
Meta-analysis of major outcomes high-flow nasal cannula as apneic oxygenation during endotracheal intubation in critically ill patients in the intensive care unit: Incidence of (**A**) severe hypoxemia (peripheral capillary oxygen saturation [SpO2] < 80%), (**B**) lowest oxygen saturation during intubation, (**C**) intensive care unit length of stay, (**D**) in-hospital mortality.

The incidence of severe hypoxemia during endotracheal intubation was examined in all seven studies^11–13,32–35^. Our analysis revealed that the incidence of severe hypoxemia did not differ between the HFNC and the standard of care groups during endotracheal intubation with the random-effects model; however, there was moderate heterogeneity among the studies (RD = −0.05, 95% CI −0.11 to 0.01; I^2^ = 38%, *P* = 0.14; Fig. 2A).

In the meta-analysis of all seven studies that evaluated lowest oxygen saturation (501 and 455 patients in the HFNC and the standard of care groups, respectively)^11–13,32–35^, the mean lowest oxygen saturation during endotracheal intubation did not differ significantly between the HFNC and the standard of care groups with the random-effects model and exhibited high heterogeneity among the studies (MD = 2.04, 95% CI = −0.17 to 4.26; I^2^ = 68%, *P* < 0.01; Fig. 2B).

In the meta-analysis of the five studies examining the length of ICU stay (430 and 385 patients in the HFNC and the standard of care groups, respectively)^12,13,32,33,35^, the patients in the HFNC group had significantly shorter ICU stays than those in the standard of care group with the random-effects model (MD = −1.80, 95% CI = −2.98 to −0.63; I^2^ = 17%, *P* = 0.31; Fig. 2C).

In the meta-analysis of the six studies examining in-hospital mortality (481 and 435 patients in the HFNC and the standard of care groups, respectively)^11–13,32,33,35^, there was no significant difference in the in-hospital mortality between the HFNC and the standard of care groups with the random-effects model (RD = −0.04, 95% CI = −0.10 to 0.01; I^2^ = 0%, *P* = 0.81; Fig. 2D).

HFNC, high-flow nasal cannula; RD, risk difference; MD, mean difference; CI, confidence interval; I^2^, I square statistic; X^2^, Cochran’s Q test.

### Minor outcomes (Supplementary Information 7)

There was no significantly difference between the HFNC and the standard of care groups with regard to the minor outcomes including the incidence of mild hypoxemia (5 studies, n = 815 patients; RD = −0.03; 95% CI = −0.10 to 0.03)^12,13,32,33,35^, the incidence of life-threatening hypoxemia (5 studies, n = 815 patients; RD = −0.01, 95% CI −0.05 to 0.03)^12,13,32,33,35^, first-pass success (5 studies, n = 671 patients; RD = 0.01, 95% CI = −0.05 to 0.06)^12,13,32–34^, duration of mechanical ventilation (5 studies, n = 815 patients; RD = −0.56, 95% CI −1.51 to 0.40)^12,13,32,33,35^, shock (4 studies, n = 665 patients; RD = −0.04, 95% CI = −0.09 to 0.02)^12,13,32,35^, cardiovascular complications (5 studies, n =766 patients; RD = −0.03, 95% CI = −0.08 to 0.01)^11–13,32,35^, and ventilator-associated pneumonia (4 studies, n = 665 patients; RD = −0.02; 95% CI = −0.07 to 0.03)^12,13,32,35^.

### Meta-regression, subgroup analyses and sensitivity analysis

The meta-regression analysis examined the relationship of the following nine variables (publication year, study country, sex, mean age, BMI, SAPS II, PaO_2_/FiO_2_ ratio, duration of intubation, and proceduralist expertise) and two major outcomes (severe hypoxemia and mean lowest oxygen saturation during endotracheal intubation) (Table 2).

**Table 2:** Meta-regression analysis of heterogeneity for severe hypoxemia and the mean lowest oxygen saturation during intubation.

Moderators	Variables	Study Number (N)	RR_interaction_ (95% CI)	P-value	Cochran Q/df	I^2^ (%)
Severe hypoxemia (SpO_2_ < 80%)	Publish Year	7	1.089 (0.763 to 1.555)	0.6372	2.27	55.87%
	Study Country	7	1.115 (0.508 to 2.446)	0.7855	2.60	61.60%
	Sex	7	0.135 (0.001 to 30.484)	0.4693	2.82	64.58%
	Mean Age	7	1.210 (0.969 to 1.511)	0.0921	1.47	32.08%
	BMI	6	1.198 (0.763 to 1.881)	0.4334	2.62	61.87%
	SAPS II	6	1.043 (0.936 to 1.163)	0.4414	2.40	58.32%
	PaO_2_ /FiO_2_ ratio	5*	0.993 (0.987 to 0.999)	0.0162*	1.12	10.52%
	Duration of intubation	6	0.994 (0.980 to 1.008)	0.4090	1.65	39.27%
	Proceduralist expertise	7	1.702 (0.588 to 4.932)	0.3270	1.97	49.25%
Lowest SpO_2_ (During intubation)	Publish Year	7	0.343 (0.084 to 1.396)	0.1352	2.52	60.37%
	Study Country	7	1.323 (0.016 to 108.115)	0.9008	3.68	72.85%
	Sex	7	0.019 (0.000 to 16963680.082)	0.7057	3.27	69.44%
	Mean Age	7	0.277 (0.065 to 1.177)	0.0820	2.70	62.99%
	BMI	6	0.444 (0.100 to 1.965)	0.2844	2.16	53.73%
	SAPS II	6	0.831 (0.416 to 1.662)	0.6015	4.57	78.13%
	PaO_2_ /FiO_2_ ratio	5*	1.004 (0.964 to 1.045)	0.8469	3.42	70.74%
	Duration of intubation	6	1.035 (0.988 to 1.084)	0.1460	1.36	26.36%
	Proceduralist expertise	7	0.190 (0.001 to 32.659)	0.5272	3.45	71.04%

BMI, Body Mass Index; SAPS II, Simplified Acute Physiology Score; Proceduralist expertise: trainee versus expert.

RR_interaction_ = interaction effect calculated by meta-regression, positive direction indicates that possible moderators might strengthen the treatment success rate in high-flow nasal cannula compared with standard of care.

P-value = The significant level was set as 0.05; Asterisks (*) = indicates statistical significance.

The meta-regression analysis showed no difference in the risk ratio of interactions of the incidence of severe hypoxemia with overall variables except PaO_2_/FiO_2_ ratio. We found a borderline significant benefit that the incidence of severe hypoxemia was lower in the patients with high PaO_2_/FiO_2_ ratio undergoing oxygen therapy with HFNC during endotracheal intubation compared to those under standard of care (risk ratio for interaction = 0.993, 95% CI = 0.987 to 0.999; I^2^ = 10.52%; Table 2). Moreover, there was no difference in the interaction of all variables with mean lowest oxygen saturation during endotracheal intubation (Table 2).

**Figure 3:**
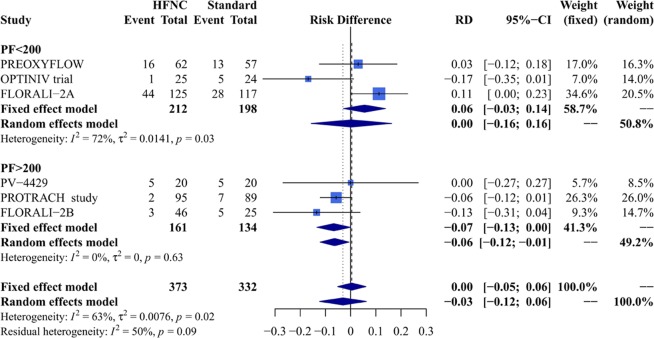
Subgroup analysis of outcomes between high-flow nasal cannula therapy and standard of care in studies investigating severe hypoxemia (SpO_2_ < 80%): The included patients were categorized by PaO_2_/FiO_2_ ratio (mild hypoxemia, PaO_2_/FiO_2_ ratio > 200 mmHg; severe-to-moderate hypoxemia, PaO_2_/FiO_2_ ratio ≤ 200 mmHg). Outcome analyses were performed using risk difference (RD) with related 95% confidence intervals (95% CI).

Based on the meta-regression analysis showing a significant interaction between severe hypoxemia and PaO_2_/FiO_2_ ratio, we performed subgroup analysis with the following clinical cut-off values for the PaO_2_/FiO_2_ ratio according to the Berlin definition of acute respiratory distress syndrome^36^ and the study by Frat *et al*.^13^): the patients with mild hypoxemia (PaO_2_/FiO_2_ ratio > 200 mmHg) and those with moderate-to-severe hypoxemia (PaO_2_/FiO_2_ ratio ≤ 200 mmHg). The analysis revealed that the benefit of HFNC in the incidence of severe hypoxemia was significantly lower among the patients with mild hypoxemia (RD, −0.06; 95% CI, −0.12 to −0.01; I^2^ = 0%; Fig. 3).

**Figure 4:**
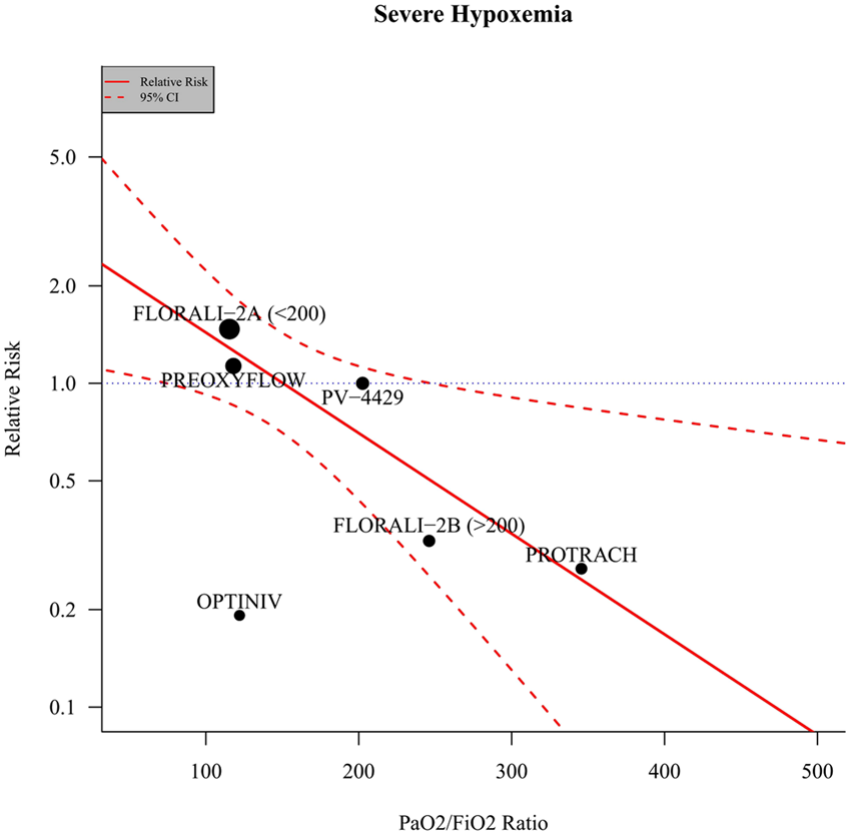
Meta-regression plot of PaO_2_/FiO_2_ ratio: Meta-regression analysis showing a linear relationship between the reduction in the incidence risk ratio of severe hypoxemia and the increase in PaO_2_/FiO_2_ ratio. As the cut-off PaO_2_/FiO_2_ ratio reached approximately 250, the upper 95% CI of severe hypoxemia incidence risk ratio equaled to 1 and then the incidence of severe hypoxemia decreased. Circles indicate incidence risk ratios of severe hypoxemia in individual studies, and bubble size is proportional to the precision of individual studies.

In the linear meta-regression interaction analysis, PaO_2_/FiO_2_ ratio significantly modified the incidence of severe hypoxemia in a linear trend. Furthermore, the risk ratio of severe hypoxemia decreased accompanied with increasing baseline PaO_2_/FiO_2_ ratios. As the cut-off PaO_2_/FiO_2_ ratio reached approximately 250, the upper 95% CI of severe hypoxemia incidence risk ratio equaled to 1 and then the incidence of severe hypoxemia decreased (Fig. 4). Overall, these results indicated that the benefit of HFNC in reducing the incidence of severe hypoxemia decreased in a linear fashion with increasing PaO_2_/FiO_2_ ratio.

In the subgroup analysis of flow rate of high-flow nasal cannula as apneic oxygenation equal to 60 L/minutes or lower than 60 L/minutes, the result revealed that the incidence of severe hypoxemia did not differ in both subgroups. (Supplementary Information 8) We also did a sensitivity analysis, altering the choice of studies to remove the non-randomized trial^11^, and the results did no change substantially. (Supplementary Information 9).

FLORALI-2A = participant stratification in FLORALI-2 study with mild hypoxemia (PaO_2_/FiO_2_ ratio > 200 mmHg)

FLORALI-2B = participant stratification in FLORALI-2 study with severe-to-moderate hypoxemia (PaO_2_/FiO_2_ ratio ≤ 200 mmHg)

PF, PaO_2_/FiO_2_ ratio; HFNC, high-flow nasal cannula; RD, risk difference; CI, confidence interval; I^2^, I square statistic; X^2^, Cochran’s Q test.

FLORALI-2A = participant stratification in FLORALI-2 study with mild hypoxemia (PaO_2_/FiO_2_ ratio > 200 mmHg)

FLORALI-2B = participant stratification in FLORALI-2 study with severe-to-moderate hypoxemia (PaO_2_/FiO_2_ ratio ≤ 200 mmHg)

CI, confidence interval; PF, PaO_2_/FiO_2_ ratio.

## Discussion

In this systematic review and meta-analysis investigating the efficacy of HFNC therapy as apneic oxygenation during endotracheal intubation in critically ill patients in the ICU setting, the available evidence suggested that HFNC as apneic oxygenation significantly reduced the length of ICU stay and that HFNC as apneic oxygenation was not inferior to standard of care oxygen therapy in the incidence of severe hypoxemia, mean lowest oxygen saturation during intubation, in-hospital mortality, and other minor outcomes. Critically, HFNC as apneic oxygenation was effective in reducing the incidence of severe hypoxemia during intubation in patients with mild hypoxemia defined by a PaO_2_/FiO_2_ ratio of >200 mmHg.

HFNC was initially utilized as an alternative breathing support for premature infants to maintain positive airway pressure^37–39^; however, there is a propensity to use HFNC therapy in adults with respiratory distress^8,40,41^. In the latest meta-analysis conducted by Zhu and colleague^40^, HFNC had significantly effect on reducing post-extubation respiratory failure rate, respiratory rates, and increasing PaO_2_, comparing with conventional oxygen therapy in patients after planned extubation. In most of studies^42–44^, HFNC was compared with standard of care to demonstrate that this approach was able to improve oxygenation. Despite several potentially physiological advantages of HFNC, there is no clear evidence of efficacy for HFNC as apneic oxygenation during endotracheal intubation. A number of RCTs provided conflicting results regarding the efficacy of HFNC as apneic oxygenation during intubation^12,13,32–35^. The current meta-analysis revealed that the incidence of severe hypoxemia was comparable between HFNC therapy and standard of care.

In the FLORALI-2 study by Frat *et al*.^13^, patients with acute respiratory distress syndrome were stratified by the PaO_2_/FiO_2_ ratio. The trial revealed that apneic oxygenation with HFNC during endotracheal intubation increased the incidence of severe hypoxemia after adjustment for PaO_2_ in patients with moderate-to-severe hypoxemia. Similarly, Simon *et al*.^45^ uncovered that the oxygen level during bronchoscopy was significantly lower in patients with moderate-to-severe hypoxemia receiving oxygen via HFNC than those receiving oxygen via noninvasive ventilation. In our subgroup analysis, we found a reduction in the incidence of severe hypoxemia in patients with mild hypoxemia among those receiving HFNC (number needed to treat = 16.7). Furthermore, in the linear meta-regression interaction analysis, we observed that the trend of reduction in hypoxemia incidence followed the reduction in baseline PaO_2_/FiO_2_ ratio in patients receiving HFNC therapy. Therefore, we propose that optimal oxygen delivery strategy should be based on the patient; by the same token, HFNC might be beneficial for apneic oxygenation in patients with mild hypoxemia.

In a meta-analysis, e Silva *et al*.^7^ found that apneic oxygenation during endotracheal intubation reduced the length of ICU stay. Notably, the inclusion of a variety of ventilation approaches for apneic oxygenation during intubation might lead to a flawed conclusion. Contrariwise, the current meta-analysis focusing on the utility of HFNC was in agreement with previous studies by revealing that providing supplemental oxygen with HFNC during intubation might be associated with a shorter ICU stay. Previous studies^11–13,32,35^ reported conflicting results with respect to cardiovascular complications including shock, arrhythmia, and cardiac death in association with HFNC. Our data showed that the incidence rates of peri-intubation shock and arrhythmia were lower during apneic oxygenation with HFNC, albeit without statistical significance.

Several ongoing RCTs are currently evaluating the efficacy of HFNC as apneic oxygenation during invasive procedures in various fields including diagnostic, emergency, and critical care medicine^46–48^. Pre- and Apneic High-Flow Oxygenation for RApid Sequence Intubation in The Emergency Department (Pre-AeRATE)^47^ is a multi-center RCT, aiming to elucidate whether HFNC improves oxygen levels during endotracheal intubation, thereby reducing the risk of hypoxemia during rapid sequence intubation. The findings of the study might provide more concrete evidence regarding the benefits and adverse events associated with HFNC.

The current meta-analysis has several limitations. First, although we focused on one approach, HFNC, the included studies employed protocols that differed in certain aspects such as different oxygen flow rates, which might have contributed to the substantial heterogeneity observed in our analyses. Second, the clinical heterogeneity of the included individuals was a critical confounding factor that should be considered. Third, the effect of HFNC as apneic oxygenation might not be isolated due to the different interventions of pre-oxygenation. Last, there was a paucity of large clinical trial to evaluate the true association between PaO_2_/FiO_2_ ratio and severe hypoxemia. As a result, a cautious approach in interpreting the results of this meta-analysis is warranted.

## Conclusion

As an oxygen delivery strategy, HFNC was noninferior to standard of care during apneic oxygenation when initiated at the time of endotracheal intubation. The meta-analysis suggested that HFNC oxygen therapy as apneic oxygenation might be beneficial by lowering the incidence of severe hypoxemia in patients with mild hypoxemia (PaO_2_/FiO_2_ ratio > 200 mmHg). Furthermore, utilizing HFNC during endotracheal intubation might be associated with a shorter ICU stay. Despite these striking findings, there is a need for further research focusing on distinguishing populations that might reap the most benefits from this approach.

## Supplementary information

Supplementary information.
