# Supplementary material for: High-flow nasal cannula therapy as apneic oxygenation during endotracheal intubation in critically ill patients in the intensive care unit: a systematic review and meta-analysis
Source: Sci Rep. 2020 Feb 26;10:3541. doi: 10.1038/s41598-020-60636-9 (PMC7044442; doi:10.1038/s41598-020-60636-9)
Supplement: Supplementary file 1 — Supplementary information. [file 41598_2020_60636_MOESM1_ESM.pdf]

Supplementary Information

High-flow nasal cannula therapy as apneic oxygenation during endotracheal intubation in critically ill patients in the intensive care unit: a systematic review and meta-analysis

Hong-Jie Jhou MD[1], Po-Huang Chen MD[2#], Chin Lin PhD[3,4], Li-Yu Yang MD[5], Cho-Hao Lee MD[6*+], Chung-Kan Peng, MD, PhD[7*+]

Contents

Supplementary Information 1. Search strategy

Supplementary Information 2. PRISMA Checklist

Supplementary Information 3. MOOSE Checklist

Supplementary Information 4. Assessment of risk of bias

Supplementary Information 5. Reference list of full-text screening studies

Supplementary Information 6. Summary of major findings

Supplementary Information 7. Meta-analysis of minor outcomes

Supplementary Information 8. Subgroup analysis of different flow rate of high-flow nasal cannula

Supplementary Information 9. Sensitivity analysis

Information 1. Search Strategy

Ovid-Medline

#1 exp "intubation"/ OR exp "laryngoscopy"

#2 ("intubat*" OR "laryngoscop*").mp

#3 exp "cannula"/

#4 ("high flow" OR "HFNO" OR "HFNOT" OR "HFNC" OR "HFNCT" OR "Transnasal Humidified Rapid Insufflation Ventilatory Exchange" OR "THRIVE" OR "nasal cannula*" OR "nasal prong*").mp

#5 1 or 2

#6 3 or 4

#7 5 and 6

Cochrane Library

#1 [mh "intubation"] OR [mh "laryngoscopy"]

#2 ("intubat*" OR "laryngoscop*"):ti,ab,kw

#3 [mh "cannula"]

#4 ("high flow" OR "HFNO" OR "HFNOT" OR "HFNC" OR "HFNCT" OR "Transnasal Humidified Rapid Insufflation Ventilatory Exchange" OR "THRIVE" OR "nasal cannula*" OR "nasal prong*"):ti,ab,kw

#5 #1 or #2

#6 #3 or #4

#7 #5 and #6

Embase

#1 "respiratory tract intubation" /exp OR " laryngoscopy " /exp

#2 ("intubat*" OR "laryngoscop*"):ti,ab,de,kw

#3 "nasal cannula" /exp

#4 ("high flow" OR "HFNO" OR "HFNOT" OR "HFNC" OR "HFNCT" OR "Transnasal Humidified Rapid Insufflation Ventilatory Exchange" OR "THRIVE" OR "nasal cannula*" OR "nasal prong*"):ti,ab,de,kw

#5 #1 or #2

#6 #3 or #4

#7 #5 and #6

Information 2 PRIMSA checklist

Section/topic	Item No	Checklist item	Reported on page No
**Title**			
Title	1	Identify the report as a systematic review, meta-analysis, or both	1
**Abstract**			
Structured summary	2	Provide a structured summary including, as applicable, background, objectives, data sources, study eligibility criteria, participants, interventions, study appraisal and synthesis methods, results, limitations, conclusions and implications of key findings, systematic review registration number	1
**Introduction**			
Rationale	3	Describe the rationale for the review in the context of what is already known	1-2
Objectives	4	Provide an explicit statement of questions being addressed with reference to participants, interventions, comparisons, outcomes, and study design (PICOS)	1-2
**Methods**			
Protocol and registration	5	Indicate if a review protocol exists, if and where it can be accessed (such as web address), and, if available, provide registration information including registration number	2
Eligibility criteria	6	Specify study characteristics (such as PICOS, length of follow-up) and report characteristics (such as years considered, language, publication status) used as criteria for eligibility, giving rationale	2
Information sources	7	Describe all information sources (such as databases with dates of coverage, contact with study authors to identify additional studies) in the search and date last searched	2
Search	8	Present full electronic search strategy for at least one database, including any limits used, such that it could be repeated	Supplementary Information 1.
Study selection	9	State the process for selecting studies (that is, screening, eligibility, included in systematic review, and, if applicable, included in the meta-analysis)	2
Data collection process	10	Describe method of data extraction from reports (such as piloted forms, independently, in duplicate) and any processes for obtaining and confirming data from investigators	2
Data items	11	List and define all variables for which data were sought (such as PICOS, funding sources) and any assumptions and simplifications made	2

Section/topic	Item No	Checklist item	Reported on page No
Risk of bias in individual studies	12	Describe methods used for assessing risk of bias of individual studies (including specification of whether this was done at the study or outcome level), and how this information is to be used in any data synthesis	2
Summary measures	13	State the principal summary measures (such as risk ratio, difference in means).	3
Synthesis of results	14	Describe the methods of handling data and combining results of studies, if done, including measures of consistency (such as I^2 statistic) for each meta-analysis	3
Risk of bias across studies	15	Specify any assessment of risk of bias that may affect the cumulative evidence (such as publication bias, selective reporting within studies)	3
Additional analyses	16	Describe methods of additional analyses (such as sensitivity or subgroup analyses, meta-regression), if done, indicating which were pre-specified	3
**Results**			
Study selection	17	Give numbers of studies screened, assessed for eligibility, and included in the review, with reasons for exclusions at each stage, ideally with a flow diagram	3
Study characteristics	18	For each study, present characteristics for which data were extracted (such as study size, PICOS, follow-up period) and provide the citations	4, Table 1
Risk of bias within studies	19	Present data on risk of bias of each study and, if available, any outcome-level assessment (see item 12).	Supplementary Information 4.
Results of individual studies	20	For all outcomes considered (benefits or harms), present for each study (a) simple summary data for each intervention group and (b) effect estimates and confidence intervals, ideally with a forest plot	5-8
Synthesis of results	21	Present results of each meta-analysis done, including confidence intervals and measures of consistency	5-8
Risk of bias across studies	22	Present results of any assessment of risk of bias across studies (see item 15)	5-8
Additional analysis	23	Give results of additional analyses, if done (such as sensitivity or subgroup analyses, meta-regression) (see item 16)	5-8

Section/topic	Item No	Checklist item	Reported on page No
**Discussion**			
Summary of evidence	24	Summarise the main findings including the strength of evidence for each main outcome; consider their relevance to key groups (such as health care providers, users, and policy makers)	9
Limitations	25	Discuss limitations at study and outcome level (such as risk of bias), and at review level (such as incomplete retrieval of identified research, reporting bias)	9
Conclusions	26	Provide a general interpretation of the results in the context of other evidence, and implications for future research	9
**Funding**			
Funding	27	Describe sources of funding for the systematic review and other support (such as supply of data) and role of funders for the systematic review	11

Information 3 MOOSE Checklist

Item No	Recommendation	Reported on Page No
Reporting of background should include		
1	Problem definition	1
2	Hypothesis statement	1
3	Description of study outcome(s)	1
4	Type of exposure or intervention used	1
5	Type of study designs used	1
6	Study population	1
Reporting of search strategy should include		
7	Qualifications of searchers (eg, librarians and investigators)	2
8	Search strategy, including time period included in the synthesis and keywords	2
9	Effort to include all available studies, including contact with authors	2
10	Databases and registries searched	2
11	Search software used, name and version, including special features used (eg, explosion)	Manual
12	Use of hand searching (eg, reference lists of obtained articles)	2
13	List of citations located and those excluded, including justification	2
14	Method of addressing articles published in languages other than English	2
15	Method of handling abstracts and unpublished studies	2
16	Description of any contact with authors	2
Reporting of methods should include		
17	Description of relevance or appropriateness of studies assembled for assessing the hypothesis to be tested	2
18	Rationale for the selection and coding of data (eg, sound clinical principles or convenience)	Supplementary Information 1.
19	Documentation of how data were classified and coded (eg, multiple raters, blinding and interrater reliability)	2-3
20	Assessment of confounding (eg, comparability of cases and controls in studies where appropriate)	2-3
21	Assessment of study quality, including blinding of quality assessors, stratification or regression on possible predictors of study results	2-3
22	Assessment of heterogeneity	3
23	Description of statistical methods (eg, complete description of fixed or random effects models, justification of whether the chosen models account for predictors of study results, dose-response models, or cumulative meta-analysis) in sufficient detail to be replicated	3
24	Provision of appropriate tables and graphics	Tables 1-2, Figs 1-4

Item No	Recommendation	Reported on Page No
Reporting of results should include		
25	Graphic summarizing individual study estimates and overall estimate	Table 1, Fig 2
26	Table giving descriptive information for each study included	Table 1
27	Results of sensitivity testing (eg, subgroup analysis)	Table 2, Fig 3-4
28	Indication of statistical uncertainty of findings	6-8
Reporting of discussion should include		
29	Quantitative assessment of bias (eg, publication bias)	7
30	Justification for exclusion (eg, exclusion of non-English language citations)	2
31	Assessment of quality of included studies	Supplementary Information 4.
Reporting of conclusions should include		
32	Consideration of alternative explanations for observed results	9
33	Generalization of the conclusions (ie, appropriate for the data presented and within the domain of the literature review)	9
34	Guidelines for future research	9
35	Disclosure of funding source	11

From: Stroup DF, Berlin JA, Morton SC, et al, for the Meta-analysis Of Observational Studies in Epidemiology (MOOSE) Group. Meta-analysis of Observational Studies in Epidemiology. A Proposal for Reporting. *JAMA*. 2000;283(15):2008-2012. doi: 10.1001/jama.283.15.2008.

Information 4 Assessment of risk of bias

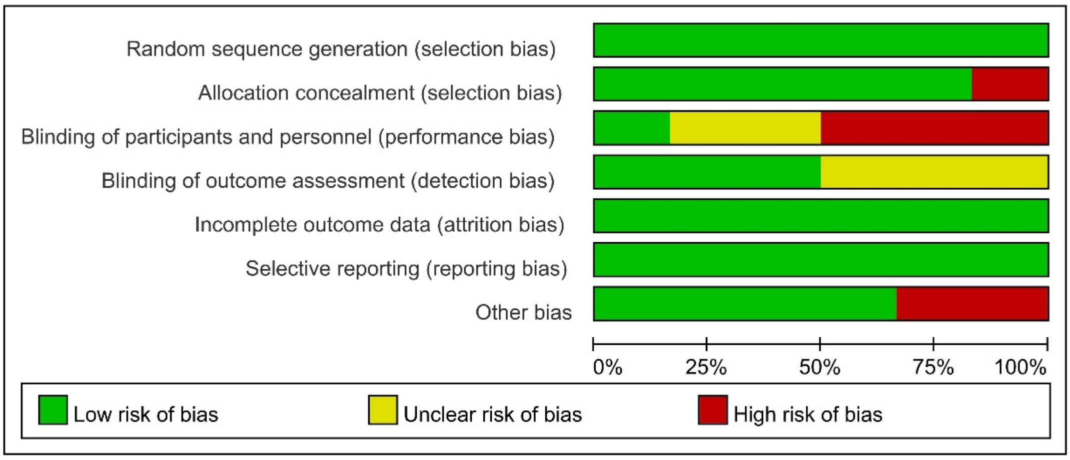

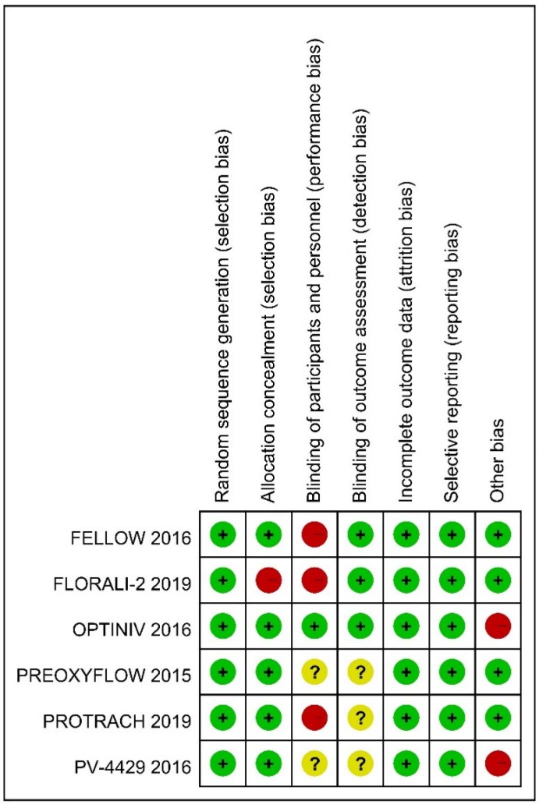

Newcastle-Ottawa Scale quality assessment scale for cohort studies

First author, year	Representativeness of the exposed cohort	Selection of the nonexposed cohort	Ascertainment of exposure	Demonstration that outcome of interest was not present at start of study	Comparability of cohorts on the basis of the design or analysis	Assessment of outcome	Was follow-up long enough for outcomes to occur	Adequacy of follow up of cohorts
Miguel-Montanes, 2015	*	*	*	*	**		*	*

Information 5 Reference list of full-text screening studies

Included studies **(No.1 to No.7)**

1. Frat, J.P., et al., *Non-invasive ventilation versus high-flow nasal cannula oxygen therapy with apnoeic oxygenation for preoxygenation before intubation of patients with acute hypoxaemic respiratory failure: a randomised, multicentre, open-label trial.* Lancet Respir Med, 2019. **7**(4): p. 303-312.
2. Guitton, C., et al., *Nasal high-flow preoxygenation for endotracheal intubation in the critically ill patient: a randomized clinical trial.* Intensive Care Med, 2019. **45**(4): p. 447-458.
3. Jaber, S., et al., *Apnoeic oxygenation via high-flow nasal cannula oxygen combined with non-invasive ventilation preoxygenation for intubation in hypoxaemic patients in the intensive care unit: the single-centre, blinded, randomised controlled OPTINIV trial.* Intensive Care Med, 2016. **42**(12): p. 1877-1887.
4. Miguel-Montanes, R., et al., *Use of high-flow nasal cannula oxygen therapy to prevent desaturation during tracheal intubation of intensive care patients with mild-to-moderate hypoxemia.* Critical Care Medicine, 2015. **43**(3): p. 574-583.
5. Semler, M.W., et al., *Randomized Trial of Apneic Oxygenation during Endotracheal Intubation of the Critically Ill.* American journal of respiratory and critical care medicine, 2016. **193**(3): p. 273-280.
6. Simon, M., et al., *High-flow nasal cannula versus bag-valve-mask for preoxygenation before intubation in subjects with hypoxemic respiratory failure.* Respiratory Care, 2016. **61**(9): p. 1160-1167.
7. Vourc'h, M., et al., *High-flow nasal cannula oxygen during endotracheal intubation in hypoxemic patients: a randomized controlled clinical trial.* Intensive care medicine, 2015. **41**(9): p. 1538-1548.

Excluded studies **(No.8 to No.59)**

8. *Abstracts of Posters Presented at the 2016 Annual Meeting of the International Anesthesia Research Society.* Anesthesia and Analgesia, 2016. **122**(5).
9. Baudin, F., et al., *Modalities and complications associated with the use of high-flow nasal cannula: Experience in a pediatric ICU.* Respiratory Care, 2016. **61**(10): p. 1305-1310.
10. Bhatia, A., J. Wang, and G. Weinhouse, *Outcomes of patients receiving high flow nasal cannula oxygen therapy in a medical ICU.* Critical Care Medicine, 2015. **43**(12): p. 183.
11. Caputo, N., et al., *Emergency Department use of Apneic Oxygenation Versus Usual Care During Rapid Sequence Intubation: A Randomized Controlled Trial (The ENDAO Trial).* Acad Emerg Med, 2017. **24**(11): p. 1387-1394.
12. Carroll, C.L., et al., *A regional cohort study of the treatment of critically ill children with bronchiolitis.* Journal of Asthma, 2016. **53**(10): p. 1006-1011.
13. Dempsey, T., *Initiative to improve outcomes through the use of an adult protocol for high-flow nasal cannula.* Respiration, 2017. **94**(1): p. 110.
14. Dhillon, N.K., et al., *Extubation to high-flow nasal cannula in critically ill surgical patients.* Journal of Surgical Research, 2017. **217**: p. 258-264.
15. Di Mussi, R., et al., *Effects of high flow nasal cannula oxygen on diaphragmatic electrical activity in the post extubation period.* Intensive Care Medicine Experimental, 2016. **4**.
16. Dixon, D.R. and D. Braude, *THE BARIATRIC AIRWAY. It's not all about the intubation.* Jems, 2015. **40**(8): p. 58-60.
17. Doyle, A.J., et al., *Preoxygenation and apneic oxygenation using Transnasal Humidified Rapid-Insufflation Ventilatory Exchange for emergency intubation.* J Crit Care, 2016. **36**: p. 8-12.

18. Dumas, G., et al., *Initial ventilation strategy and risk for intubation in immunocompromised patients with acute respiratory failure.* Annals of Intensive Care, 2017. **7**(1): p. 147-148.

19. Ffrench-O'carroll, R., et al., *Maintaining oxygenation with high-flow nasal cannula during emergent awake surgical tracheostomy.* British Journal of Anaesthesia, 2017. **118**(6): p. 954-955.

20. Forst, B., et al., *High flow nasal cannulae oxygen therapy reduces the rate of tracheal intubation in infants with acute lung injury.* Critical Care Medicine, 2010. **38**: p. A102.

21. Frat, J.P., et al., *Preoxygenation with non-invasive ventilation versus high-flow nasal cannula oxygen therapy for intubation of patients with acute hypoxaemic respiratory failure in ICU: The prospective randomised controlled FLORALI-2 study protocol.* BMJ Open, 2017. **7**(12).

22. Gagnon, S., et al., *Safety of high-flow nasal cannula in a pediatric intensive care unit.* Canadian Respiratory Journal, 2015. **22**(3): p. e9.

23. Hathorn, C., et al., *The hi-FLO study: A prospective open randomised controlled trial of high flow nasal cannula oxygen therapy against standard care in bronchiolitis.* Thorax, 2014. **69**: p. A38.

24. Hernandez, G., et al., *The role of nasal cannula high flow conditioned oxygen therapy on non-invasive mechanical ventilation performance in acute respiratory failure: A preliminary cohort study.* Intensive Care Medicine, 2013. **39**: p. S359.

25. Humphreys, S., et al., *Transnasal humidified rapid-insufflation ventilatory exchange (THRIVE) in children: a randomized controlled trial.* Br J Anaesth, 2017. **118**(2): p. 232-238.

26. Jaber, S., N. Molinari, and A. De Jong, *New method of preoxygenation for orotracheal intubation in patients with hypoxaemic acute respiratory failure in the intensive care unit, non-invasive ventilation combined with apnoeic oxygenation by high flow nasal oxygen: therandomised OPTINIV study protocol.* BMJ Open, 2016. **6**(8): p. e011298.

27. Kang, W.Q., et al., *Efficacy of heated humidified high-flow nasal cannula in preterm infants aged less than 32 weeks after ventilator weaning.* Chinese Journal of Contemporary Pediatrics, 2016. **18**(6): p. 488-491.

28. Kugelman, A., *Utility of high-flow nasal cannula therapy in the NICU.* Pediatric Pulmonology, 2018. **53**: p. S31-S32.

29. Miller, A.G., et al., *High-flow nasal cannula in pediatric patients: A survey of clinical practice.* Respiratory Care, 2018. **63**(7): p. 894-899.

30. Moretti, C., et al., *Nasal flow-synchronized intermittent positive pressure ventilation to facilitate weaning in very low-birthweight infants: unmasked randomized controlled trial.* Pediatr Int, 2008. **50**(1): p. 85-91.

31. Mortimer, T., et al., *Apneic Oxygenation during Rapid Sequence Intubation in Critically Ill Children.* J Pediatr Intensive Care, 2016. **5**(1): p. 28-31.

32. Napolitano, N., et al., *Noninvasive ventilation factors associated with the safety of tracheal intubation.* Critical Care Medicine, 2018. **46**: p. 642.

33. Napolitano, N., et al., *Impact of noninvasive ventilation use on safety of tracheal intubation: A multicenter study.* Critical Care Medicine, 2016. **44**(12): p. 141.

34. Ng, I., et al., *The use of Transnasal Humidified Rapid-Insufflation Ventilatory Exchange (THRIVE) for pre-oxygenation in neurosurgical patients: a randomised controlled trial.* Anaesth Intensive Care, 2018. **46**(4): p. 360-367.

35. Overmann, K.M., et al., *Apneic oxygenation to prevent oxyhemoglobin desaturation during rapid sequence intubation in a pediatric emergency department.* Am J Emerg Med, 2018.

36. Rebollo, S., et al., *Non-invasive ventilation vs. high flow nasal cannula oxygenation in pneumonia-associated acute respiratory failure. An observational retrospective study.* Intensive Care Medicine Experimental, 2018. **6**.

37. Rello, J., et al., *High-flow nasal therapy in adults with severe acute respiratory infection. A cohort study in patients with 2009 influenza A/H1N1v.* Journal of Critical Care, 2012. **27**(5): p. 434-439.

38. Riddell, A., *The Effect of Apneic Oxygenation on Reducing Hypoxemia During Rapid Sequence Induction and Intubation in the Acutely Ill or Injured.* Adv Emerg Nurs J, 2017. **39**(4): p. 309-317.

39. Riva, T., et al., *High-flow nasal cannula therapy and apnea time in laryngeal surgery.* Paediatric Anaesthesia, 2016. **26**(12): p. 1206-1208.

40. Riyapan, S. and J. Lubin, *Apneic Oxygenation May Not Prevent Severe Hypoxemia During Rapid Sequence Intubation: A Retrospective Helicopter Emergency Medical Service Study.* Air Med J, 2016. **35**(6): p. 365-368.

41. Roked, F., S. Samar, and D. Jyothish, *4-year experience of using high flow nasal cannula (HFNC) oxygen therapy on paediatric wards.* Archives of Disease in Childhood, 2018. **103**: p. A38.

42. Sahay, N., et al., *Effect of nasal oxygen supplementation during apnoea of intubation on arterial oxygen levels: A prospective randomised controlled trial.* Indian J Anaesth, 2017. **61**(11): p. 897-902.

43. Saksitthichok, B., et al., *A prospective randomized comparative study of high-flow nasal cannula oxygen and non-invasive ventilation in hypoxemic patients undergoing diagnostic flexible bronchoscopy.* Respirology, 2017. **22**: p. 241.

44. Sankaranarayanan, S., et al., *Profile of patients on high flow nasal cannula (HFNC) in PICU.* Pediatric Critical Care Medicine, 2018. **19**(6): p. 239.

45. Schlapbach, L.J., et al., *High-flow nasal cannula (HFNC) support in interhospital transport of critically ill children.* Intensive Care Medicine, 2014. **40**(4): p. 592-599.

46. Shippam, W., et al., *High-flow nasal oxygen vs. standard flow-rate facemask pre-oxygenation in pregnant patients: a randomised physiological study.* Anaesthesia, 2019. **74**(4): p. 450-456.

47. Shoemaker, M.T., et al., *High flow nasal cannula versus nasal CPAP for neonatal respiratory disease: A retrospective study.* Journal of Perinatology, 2007. **27**(2): p. 85-91.

48. Simon, M., et al., *High-flow nasal cannula oxygen versus non-invasive ventilation in patients with acute hypoxaemic respiratory failure undergoing flexible bronchoscopy - a prospective randomised trial.* Critical Care, 2014. **18**(1).

49. Sitthikarnkha, P., et al., *High-flow nasal cannula versus conventional oxygen therapy in children with respiratory distress.* Indian Journal of Critical Care Medicine, 2018. **22**(5): p. 321-325.

50. Stoever, J., et al., *Safety and efficacy of high flow nasal cannula oxygen therapy in acute hypercapnic respiratory failure: A pilot study.* American Journal of Respiratory and Critical Care Medicine, 2018. **197**(MeetingAbstracts).

51. Sztrymf, B., et al., *Beneficial effects of humidified high flow nasal oxygen in critical care patients: A prospective pilot study.* Intensive Care Medicine, 2011. **37**(11): p. 1780-1786.

52. Tan, P. and A.T. Dennis, *High flow humidified nasal oxygen in pregnant women.* Anaesth Intensive Care, 2018. **46**(1): p. 36-41.

53. Tinnevelt, M., et al., *Safety and efficacy of early extubation after fontan surgery with the use of nitric oxide delivery with high flow nasal canula.* European Journal of Pediatrics, 2016. **175**(11): p. 1443.

54. Tremblay, J.A., et al., *Non-invasive administration of inhaled nitric oxide in critically ill adults and its effects on right ventricular function-a cohort study.* Canadian Journal of Anesthesia, 2018. **65**: p. S84-S86.

55. Van Beers, F., et al., *Short-term effect of humidified high nasal flow oxygen in critically ill patients.* Critical Care, 2012. **16**: p. S49.

56. West, J.R., et al., *The effect of the apneic period on the respiratory physiology of patients undergoing intubation in the ED.* Am J Emerg Med, 2017. **35**(9): p. 1320-1323.

57. Wimalasena, Y., et al., *Apneic oxygenation was associated with decreased desaturation rates during rapid sequence intubation by an Australian helicopter emergency medicine service.* Ann Emerg Med, 2015. **65**(4): p. 371-6.

58. Wittenstein, J., et al., *High-flow nasal cannula oxygen therapy in patients undergoing thoracic surgery: current evidence and practice.* Curr Opin Anaesthesiol, 2019. **32**(1): p. 44-49.

59. Xiao, L. and M. Zhang, *Bedside ultrasound assessment of lung reaeration in patients with blunt thoracic injury receiving high-flow nasal cannula therapy: A retrospective study.* Intensive Care Medicine Experimental, 2018. **6**

Ongoing studies: Clinical trial protocol **(No.60 to No.62)**

60. Ostrovsky, I. Apneic Oxygenation Including Precipitous Intubations During RSI in the ED (RAPID), <https://clinicaltrials.gov/ct2/show/NCT03694379> (2019)

61. Chua, M. T. et al. Pre- and Apnoeic high flow oxygenation for RApid sequence intubation in The Emergency department (Pre-AeRATE): study protocol for a multicentre, randomised controlled trial. Trials 20, 195, doi:10.1186/s13063-019-3305-8 (2019).

62. Vourc'h, M. et al. Preoxygenation in difficult airway management: high-flow oxygenation by nasal cannula versus face mask (the PREOPTIDAM study). Protocol for a single-centre randomised study. BMJ open 9, e025909, doi:10.1136/bmjopen-2018-025909 (2019).

Meeting abstract only **(No.63 to No.64)**

63. Ferguson C. et al. A Review of the Use of High Flow Nasal Cannula on a Respiratory Ward in a District General Hospital. Am J Respir Crit Care Med 2018;197:A5099. <https://www.atsjournals.org/doi/abs/10.1164/ajrccm-conference.2018.197.1_MeetingAbstracts.A5099#affl>

64. Guoqiang J. et al. Late Breaking Abstract - Comparison of High Flow Nasal Cannula with Noninvasive Ventilation in Facilitating Weaning COPD From Invasive Ventilation: A Prospective Randomized Controlled Study. European Respiratory Journal 2018 52: Suppl. 62, PA3338. < https://erj.ersjournals.com/content/52/suppl_62/PA3338>

Information 6 Summary of major findings

Summary of findings:

High-flow nasal cannula therapy as apneic oxygenation during endotracheal intubation in critically ill patients in the intensive care unit

Patient or population: Critically ill patients receiving endotracheal intubation

Setting: In the intensive care unit

Intervention: High-flow nasal cannula therapy

Comparison: Standard of care

Outcomes	**Anticipated absolute effects*** (95% CI)		Relative effect (95% CI)	№ of participants (studies)	Certainty of the evidence (GRADE)	Comments
	**Risk with Standard of care**	**Risk with High-flow nasal cannula therapy**				
Incidence of severe Hypoxemia (SpO2 < 80%)	193 per 1,000	**145 per 1,000** (91 to 234)	**RR 0.75** (0.47 to 1.21)	956 (7 RCTs)	⨁⨁⨁◯ MODERATE [a]	HFNC possibly achieve a small or no reduction in the incidence of severe hypoxemia during ETI.
Mean lowest oxygen saturation during ETI		MD **2.04 % higher** (0.17 to 4.26)	-	956 (7 RCTs)	⨁⨁◯◯ LOW [a,b]	HFNC possibly have a small or no increase in the oxygen saturation during ETI.
ICU length of stay		MD **1.8 days lower** (0.63 to 2.98)	-	815 (5 RCTs)	⨁⨁⨁◯ MODERATE [a]	HFNC possibly decrease the ICU length of stay.
In-hospital Mortality	354 per 1,000	**304 per 1,000** (255 to 365)	**RR 0.86** (0.72 to 1.03)	916 (6 RCTs)	⨁⨁◯◯ LOW [a,c]	HFNC possibly achieve a small or no reduction in the in-hospital mortality.

***The risk in the intervention group** (and its 95% confidence interval) is based on the assumed risk in the comparison group and the **relative effect** of the intervention (and its 95% CI). **CI:** Confidence interval; **RR:** Risk ratio; **MD:** Mean difference

GRADE Working Group grades of evidence

High certainty: We are very confident that the true effect lies close to that of the estimate of the effect

Moderate certainty: We are moderately confident in the effect estimate: The true effect is likely to be close to the estimate of the effect, but there is a possibility that it is substantially different

Low certainty: Our confidence in the effect estimate is limited: The true effect may be substantially different from the estimate of the effect

Very low certainty: We have very little confidence in the effect estimate: The true effect is likely to be substantially different from the estimate of effect

Explanations

a. Lack of allocation concealment and blinding; Include prospective cohort study

b. Substantial inconsistency (I Square = 68 %)

c. Differences in outcomes measures: Time differences in outcomes

Information 7. Meta-analysis of minor outcomes

Supplementary Table 2. Meta-analysis of minor outcomes with high-flow nasal cannula as apneic oxygenation during endotracheal intubation in critically ill patients in the intensive care unit

**Outcome or Complication during Intubation**	**No. of trials (patients)**	**Risk Difference (95% CI) Random-Effect estimate**	**Risk Difference (95% CI) Fixed-Effect estimate**	**Heterogeneity I^2(%)**	**Cochran's Q p-value**
Hypoxemia (SpO2 < 90%)	5 (815)	-0.03 (-0.10 ; 0.03)	-0.01 (-0.08 ; 0.05)	16.0%	0.31
Life-threatening hypoxemia (SpO2 < 70%)	5 (815)	-0.01 (-0.05 ; 0.03)	0.00 (-0.04 ; 0.04)	17.0%	0.30
First-pass success	5 (671)	0.01 (-0.05 ; 0.06)	0.01 (-0.05 ; 0.07)	0.0%	0.93
Duration of mechanical ventilation, days	5 (815)	-0.56 (-1.51 ; 0.40)	-0.36 (-1.02 ; 0.30)	42.0%	0.14
Shock	4 (665)	-0.04 (-0.09 ; 0.02)	-0.03 (-0.10 ; 0.03)	0.0%	0.49
Cardiovascular Complication	5 (766)	-0.03 (-0.08 ; 0.01)	-0.03 (-0.08 ; 0.03)	0.0%	0.49
Ventilation Associated Pneumonia	4 (665)	-0.02 (-0.07 ; 0.03)	-0.02 (-0.07 ; 0.04)	0.0%	0.89

CI, confidence interval; HFNC, high-flow nasal cannula; SpO_2, saturation of peripheral oxygen.

Shock = defined as systolic blood pressure < 80 mmHg or that requiring vasopressor introduction or increasing vasopressor dose by more than 30%;

Cardiovascular complication = defined as shock, arrhythmia, and cardiac arrest.

I^2, index for assessing heterogeneity, value >50% indicates a moderate to high heterogeneity; Cochran's Q P value = $P < 0.10$ as a threshold to determine heterogeneity.

Information 8. Subgroup analysis of different flow rate of high-flow nasal cannula

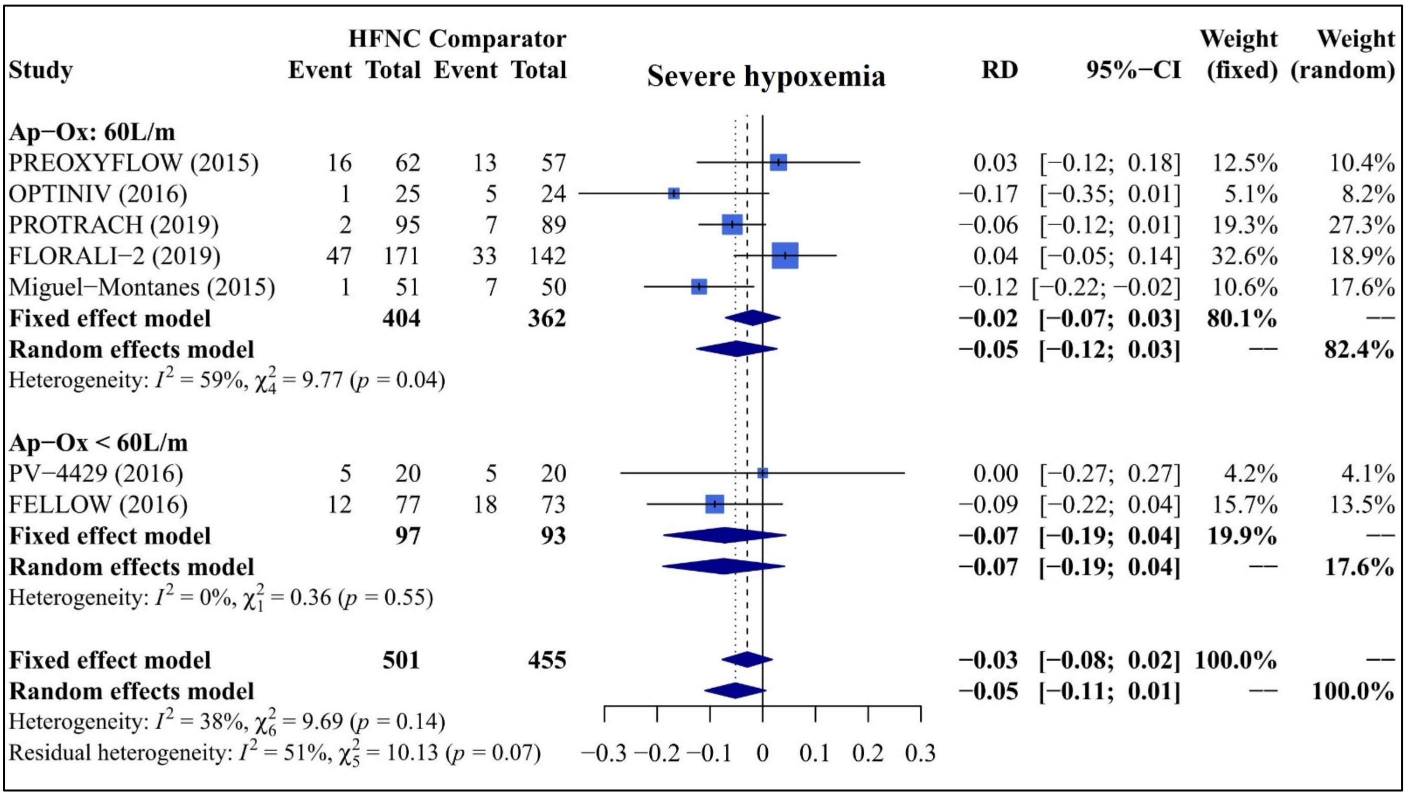

Information 8A. Subgroup analysis of outcomes between high-flow nasal cannula therapy and comparator in studies investigating severe hypoxemia (SpO2 <80%): The included patients were categorized by flow rate of high-flow nasal cannula as apneic oxygenation equal to 60 L/m or lower than 60 L/m. Outcome analyses were performed using risk difference (RD) with related 95% confidence intervals (95%CI).

Ap-Ox, Apneic oxygenation; HFNC, high-flow nasal cannula; RD, risk difference; CI, confidence interval; I^2, I square statistic; X^2, Cochran's Q P value: P < 0.10 as a threshold to determine

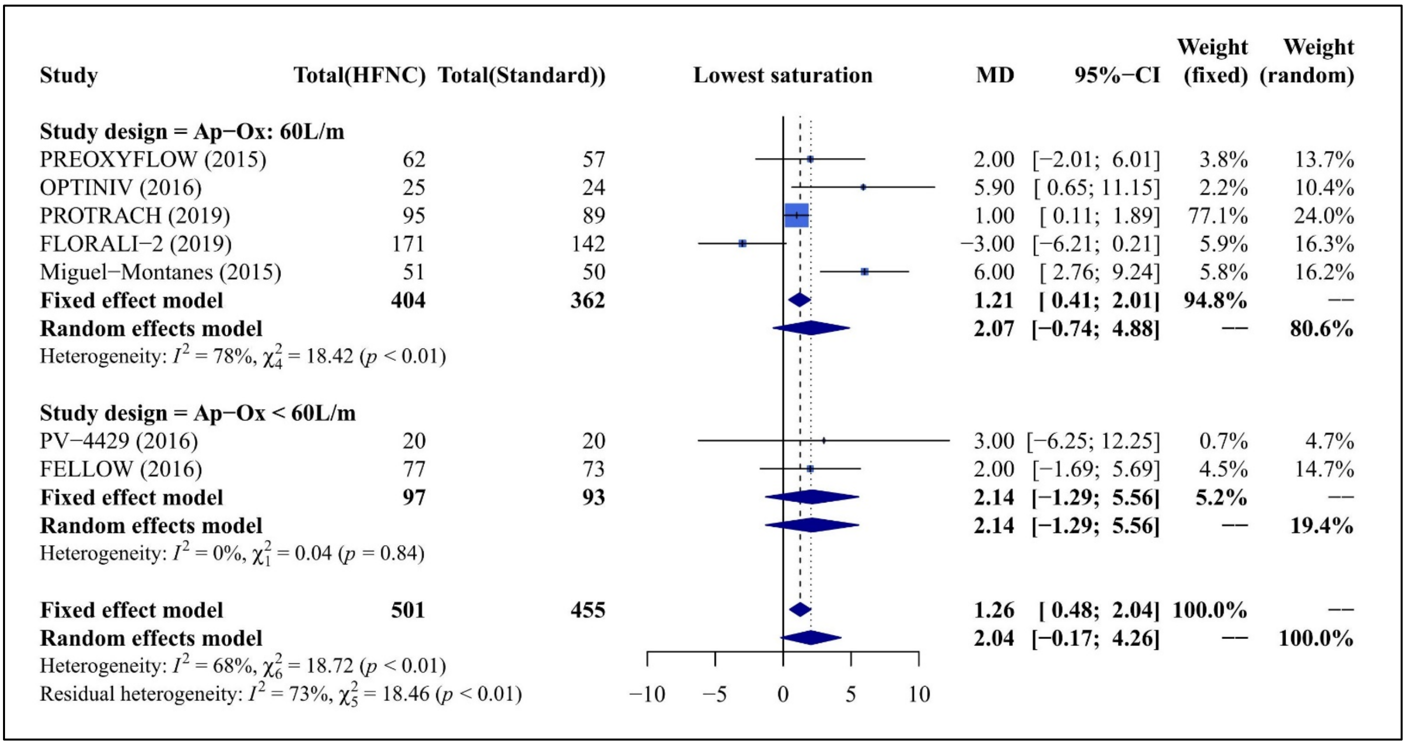

Information 8B. Subgroup analysis of outcomes between high-flow nasal cannula therapy and comparator in studies investigating mean lowest saturation during intubation: The included patients were categorized by flow rate of high-flow nasal cannula as apneic oxygenation equal to 60 L/m or lower than 60 L/m. Outcome analyses were performed using mean difference (MD) with related 95% confidence intervals (95%CI).

Ap-Ox, Apneic oxygenation; HFNC, high-flow nasal cannula; MD, mean difference; CI, confidence interval; I^2, I square statistic; X^2, Cochran's Q P value: P < 0.10 as a threshold to determine

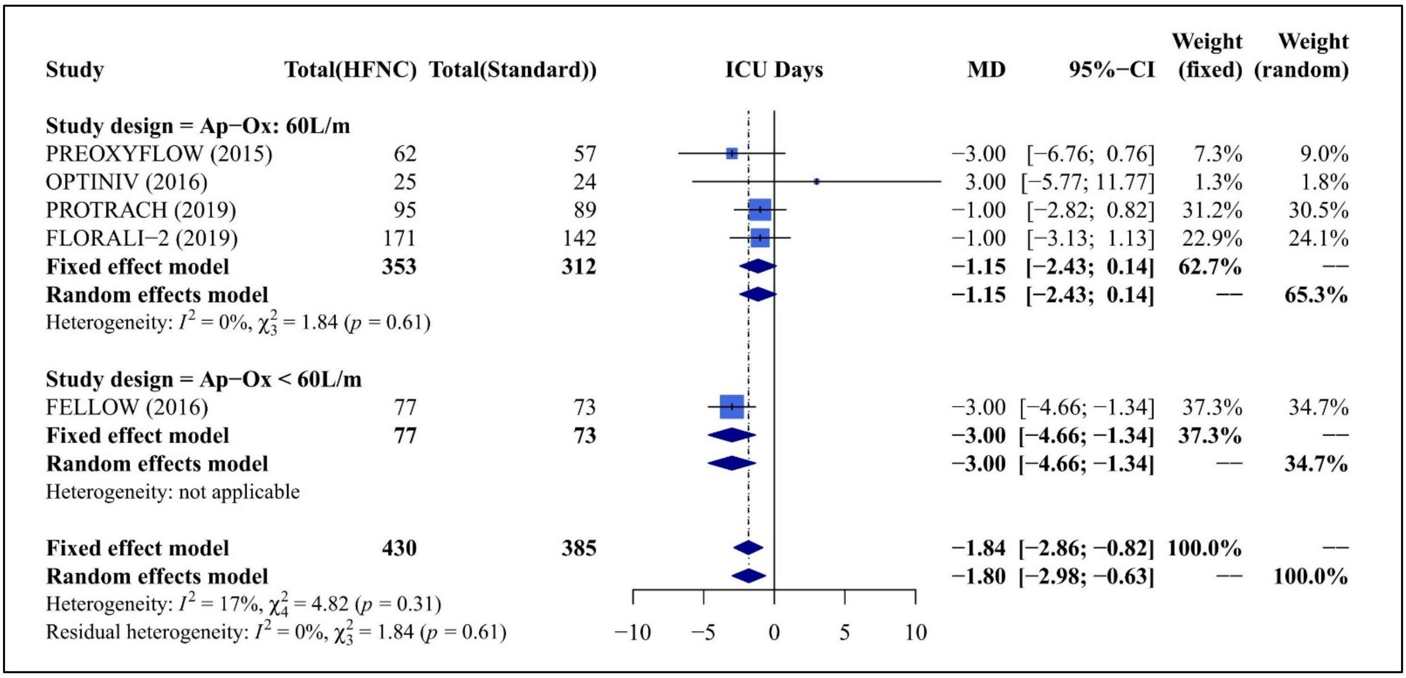

Information 8C. Subgroup analysis of outcomes between high-flow nasal cannula therapy and comparator in studies investigating length of ICU stay (days): The included patients were categorized by flow rate of high-flow nasal cannula as apneic oxygenation

equal to 60 L/m or lower than 60 L/m. Outcome analyses were performed using mean difference (MD) with related 95% confidence intervals (95%CI).

Ap-Ox, Apneic oxygenation; HFNC, high-flow nasal cannula; MD, mean difference; CI, confidence interval; I^2, I square statistic; X^2, Cochran's Q P value: P < 0.10 as a threshold to determine

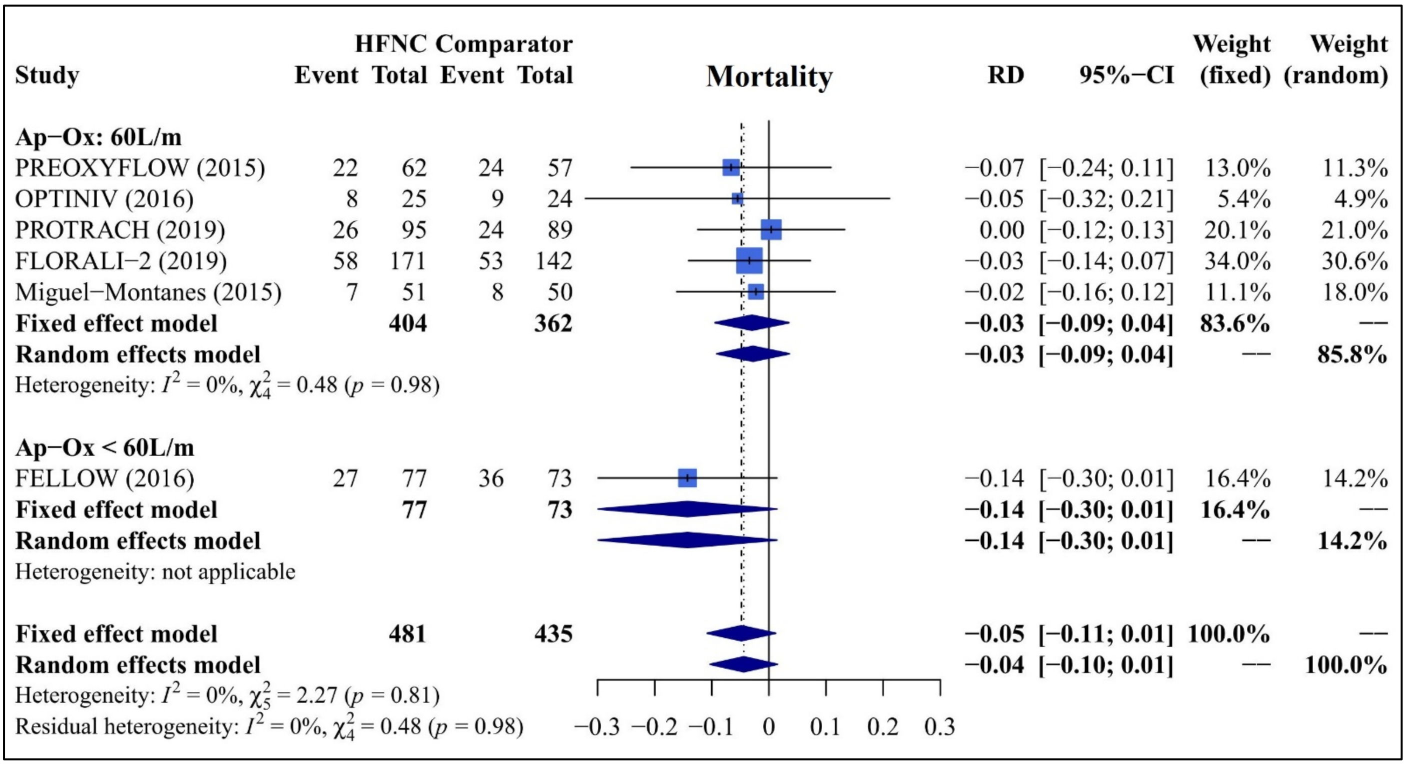

Information 8D. Subgroup analysis of outcomes between high-flow nasal cannula therapy and comparator in studies investigating: in-hospital mortality rate. The included patients were categorized by flow rate of high-flow nasal cannula as apneic oxygenation equal to 60 L/m or lower than 60 L/m. Outcome analyses were performed using risk difference (RD) with related 95% confidence intervals (95%CI).

Ap-Ox, Apneic oxygenation; HFNC, high-flow nasal cannula; RD, risk difference; CI, confidence interval; I^2, I square statistic; X^2, Cochran's Q P value: P < 0.10 as a threshold to determine

Information 9. Sensitivity analysis

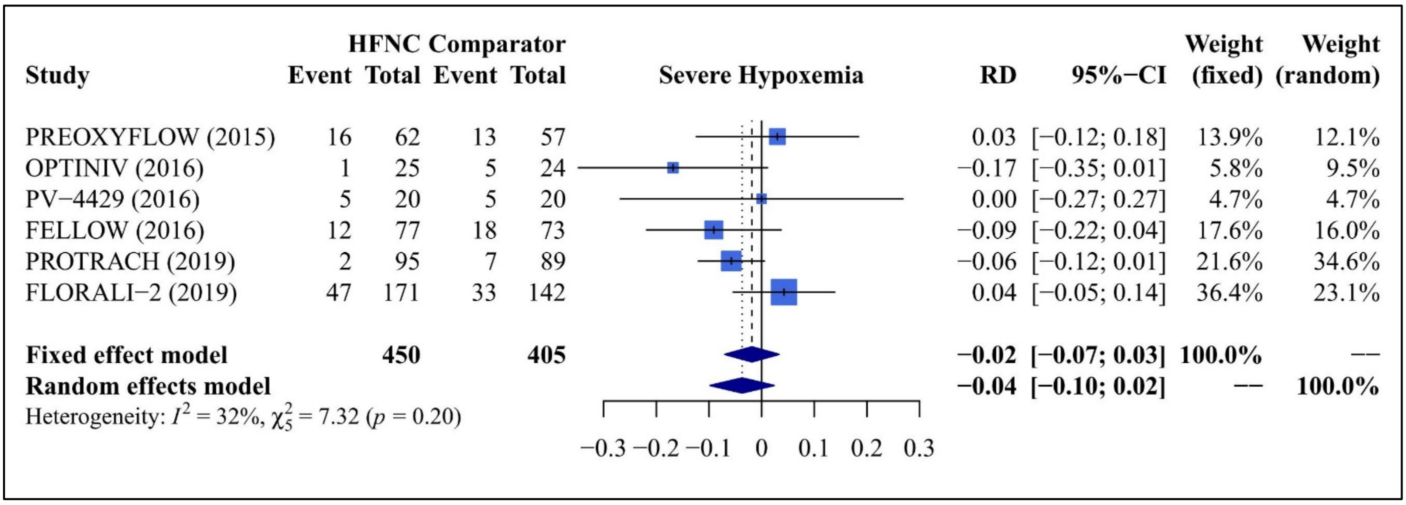

Information 9A. Sensitivity analysis of excluding study design as non-randomized study: incidence of severe hypoxemia (peripheral capillary oxygen saturation [SpO2] < 80%) HFNC, high-flow nasal cannula; RD, risk difference; CI, confidence interval; I2, I square statistic; X2, Cochran's Q test.

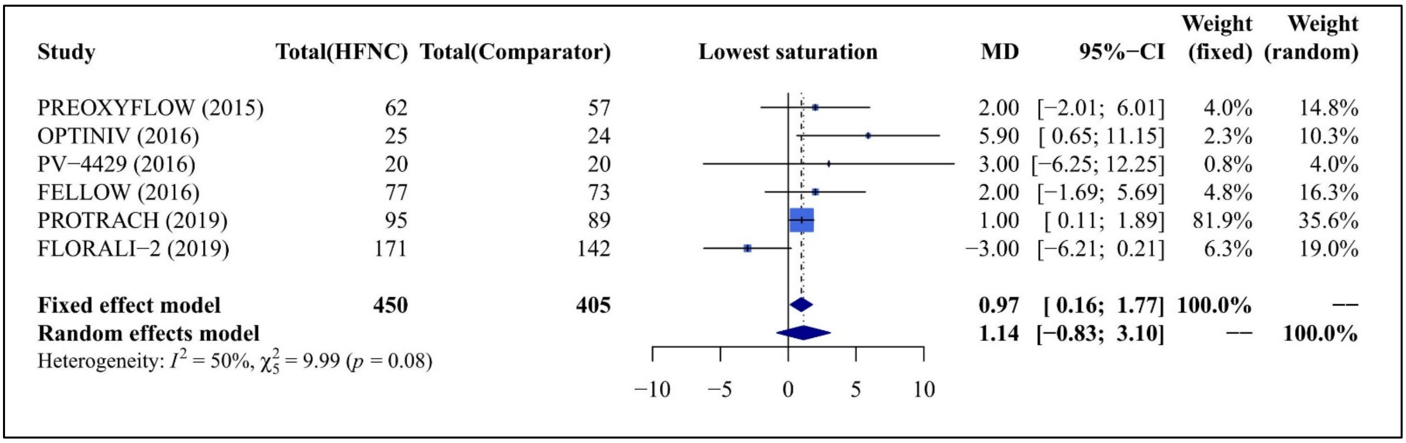

Information 9B. Sensitivity analysis of excluding study design as non-randomized study: lowest oxygen saturation during intubation

HFNC, high-flow nasal cannula; MD, mean difference; CI, confidence interval; I2, I square statistic; X2, Cochran's Q test.

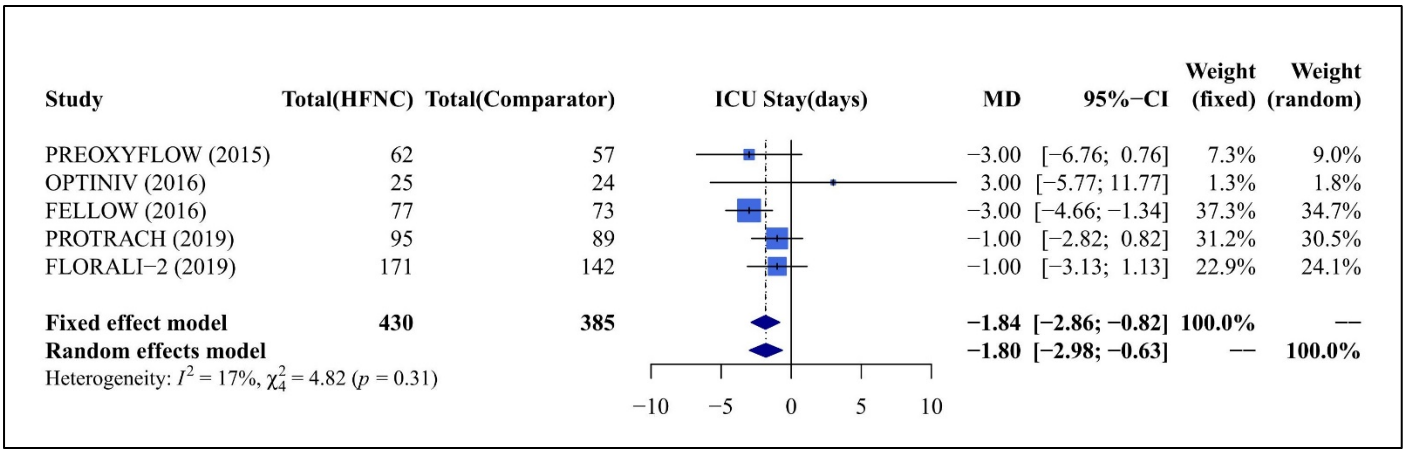

Information 9C. Sensitivity analysis of excluding study design as non-randomized study: intensive care unit length of stay

HFNC, high-flow nasal cannula; MD, mean difference; CI, confidence interval; I2, I square statistic; X2, Cochran's Q test.

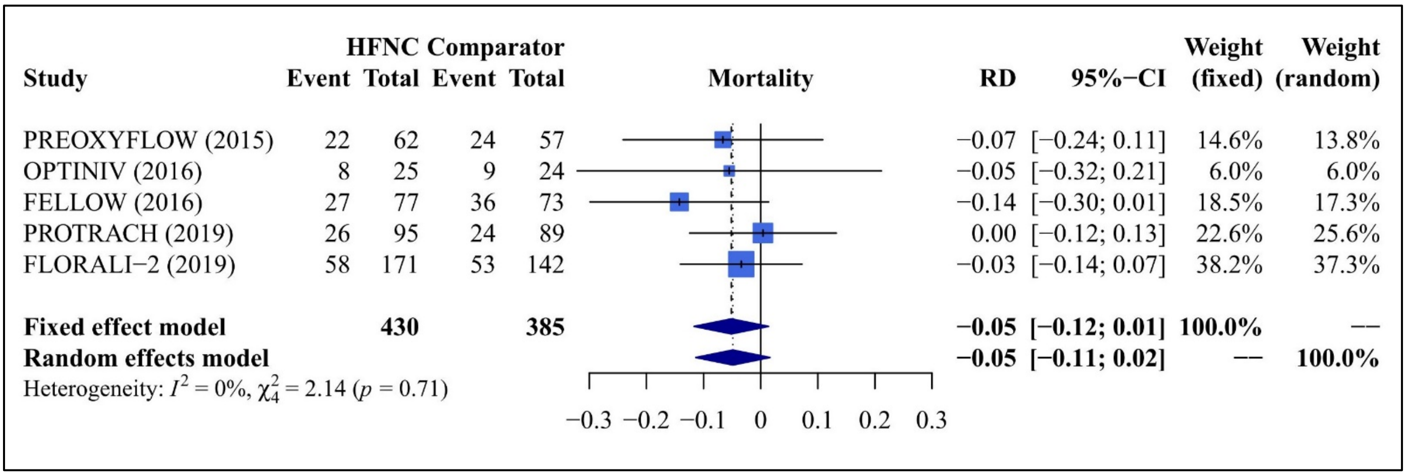

Information 9D. Sensitivity analysis of excluding study design as non-randomized study: in-hospital mortality

HFNC, high-flow nasal cannula; RD, risk difference; CI, confidence interval; I2, I square statistic; X2, Cochran's Q test.
